# Radiomics-Based Texture Analysis of ^68^Ga-DOTATATE Positron Emission Tomography and Computed Tomography Images as a Prognostic Biomarker in Adults With Neuroendocrine Cancers Treated With ^177^Lu-DOTATATE

**DOI:** 10.3389/fonc.2021.686235

**Published:** 2021-08-02

**Authors:** Charlotte Atkinson, Balaji Ganeshan, Raymond Endozo, Simon Wan, Matthew D. Aldridge, Ashley M. Groves, Jamshed B. Bomanji, Mark N. Gaze

**Affiliations:** ^1^Departments of Oncology and Nuclear Medicine, University College London Hospitals NHS Foundation Trust, London, United Kingdom; ^2^Institute of Nuclear Medicine, University College London, London, United Kingdom

**Keywords:** ^68^Ga-DOTATATE PET/CT, ^177^Lu-DOTATATE molecular radiotherapy, neuroendocrine tumor (NET), prognostic biomarker, texture analysis, radiomics

## Abstract

**Purpose:**

Neuroendocrine tumors (NET) are rare cancers with variable behavior. A better understanding of prognosis would aid individualized management. The aim of this hypothesis-generating pilot study was to investigate the prognostic potential of tumor heterogeneity and tracer avidity in NET using texture analysis (TA) of ^68^Ga-DOTATATE positron emission tomography (PET) and non-enhanced computed tomography (CT) performed at baseline in patients treated with ^177^Lu-DOTATATE. It aims to justify a larger-scale study to evaluate its clinical value.

**Methods:**

The pretherapy ^68^Ga-DOTATATE PET-CT scans of 44 patients with metastatic NET (carcinoid, pancreatic, thyroid, head and neck, catecholamine-secreting, and unknown primary NET) treated with ^177^Lu-DOTATATE were analyzed retrospectively using commercially available texture analysis research software. Image filtration extracted and enhanced objects of different sizes (fine, medium, coarse), then quantified heterogeneity by statistical and histogram-based parameters (mean intensity, standard deviation, entropy, mean of positive pixels, skewness, and kurtosis). Regions of interest were manually drawn around up to five of the most ^68^Ga-DOTATATE avid lesions for each patient. ^68^Gallium uptake on PET was quantified as SUV_max_ and SUV_mean_. Associations between imaging and clinical markers with progression-free (PFS) and overall survival (OS) were assessed using univariate Kaplan-Meier analysis. Independence of the significant univariate markers of survival was tested using multivariate Cox regression analysis.

**Results:**

Measures of heterogeneity (higher kurtosis, higher entropy, and lower skewness) on coarse-texture scale CT and unfiltered PET images predicted shorter PFS (CT coarse kurtosis: p=0.05, PET entropy: p=0.01, PET skewness: p=0.03) and shorter OS (CT coarse kurtosis: p=0.05, PET entropy: p=0.01, PET skewness p=0.02). Conventional PET parameters such as SUV_max_ and SUV_mean_ showed trends towards predicting outcome but were not statistically significant. Multivariate analysis identified that CT-TA (coarse kurtosis: HR=2.57, 95% CI=1.22–5.38, p=0.013) independently predicted PFS, and PET-TA (unfiltered skewness: HR=9.05, 95% CI=1.19–68.91, p=0.033) independently predicted OS.

**Conclusion:**

These preliminary data generate a hypothesis that radiomic analysis of neuroendocrine cancer on ^68^Ga-DOTATATE PET-CT may be of prognostic value and a valuable addition to the assessment of patients.

## Introduction

Neuroendocrine tumors (NET) are rare and heterogeneous. This variety presents challenges when making decisions about sequencing of therapies in metastatic disease. Clinical features such as primary tumor, metastatic characteristics, and prior systemic treatments have prognostic potential (1). However, they are inconsistent, and more reliable markers are needed.

Somatostatin receptor (STTR) expression on the tumor cell surface can be measured by ^68^Ga-DOTATATE positron emission tomography (PET) computed tomography (CT) hybrid-imaging scans. There is evidence that tumor avidity on ^68^Ga-DOTATATE scans may be linked to outcome in NET patients (2, 3).

De-differentiation of NET is often associated with a more aggressive phenotype. Histologic intratumor heterogeneity is a measure of this (4) but is difficult to evaluate in metastases. Functional imaging is simpler for assessment of metastatic heterogeneity and enables quantitative assessment. Texture analysis (TA) of CT and PET images, where the tumor coarseness and distribution of pixel gray-level intensity can reflect tumor heterogeneity, is one such tool (5). It uses standard non-invasive diagnostic imaging, avoiding the need for extra radiation or additional complex, invasive procedures or expense. TA has been used in various tumors including lung and esophageal cancers: higher indices of heterogeneity are associated with worse outcomes (6–8).

Could PET and CT-TA be useful, in addition to clinical factors, for prognostication in NET? With better prognostic information, management of metastatic NET could be individualized, leading to personalized selection of treatments in those most likely to benefit.

This pilot study aimed to demonstrate the prognostic potential of tumor avidity and heterogeneity in patients with NET from a single institution treated with ^177^Lutetium DOTATATE peptide receptor radionuclide therapy (PRRT). We hypothesized that tumors with higher heterogeneity scores and lower standardized uptake values (SUV) would be associated with a worse prognosis.

## Materials and Methods

### Patients

This retrospective pilot study included all NET patients aged over 18 years treated at our hospital with ^177^Lu-DOTATATE molecular radiotherapy between 2007 and 2016. It included carcinoid, pancreatic, thyroid, head and neck, catecholamine-secreting, and unknown primary NET. Neuroblastoma and central nervous system NET were excluded. Ethical approval for data collection and analysis was granted by HRA and Health and Care Research Wales (IRAS project number 204124).

For treatment, patients had to have a baseline ^68^Ga-DOTATATE PET-CT with tumor avidity greater than liver. Other criteria were creatinine <150 µmol L^−1^ or creatinine clearance >50 ml min^−1^ 1.72 m^−2^, and Eastern Cooperative Oncology Group (ECOG) performance status of 3 or less.

### Treatment

^177^Lu-DOTATATE was obtained commercially from IDB Holland (http://www.idb-holland.com/our-products/lutetium-177-lumark/- Baarle-Nassau, Netherlands). Patients were planned to receive four administrations of ^177^Lu-DOTATATE at a dose of 7.4 GBq. Those with glomerular filtration rate of <60 ml min^−1^ 1.72 m^−2^ were treated with 50% dose reduction. Premedication was with oral ondansetron and dexamethasone over 5 days. To reduce renal radiation, an intravenous infusion of amino acids (2.5% L-lysine and 2.5% L-arginine) was commenced (1 L/4 h) 30 min before administration of intravenous ^177^Lu-DOTATATE, given over 30 min. Subsequently, patients were monitored weekly for toxicities. Typically, four courses were administered at 8- to 12-week intervals; however, treatment was discontinued for disease progression or toxicity.

### Follow-Up

Patients were followed and imaged 6 monthly with ^68^Ga-DOTATATE PET-CT. The primary outcome measure was progression-free survival (PFS) as further cancer therapy might affect overall survival. PFS was calculated from the date of first ^177^Lu-DOTATATE course to disease progression of any type; classified on ^68^Ga-DOTATATE PET-CT as development of a new metastasis or a 20% increase in target lesion size (RECIST v1.1) and not increase in SUV of a target lesion. Overall survival (OS) was recorded.

### PET-CT Image Acquisition

^68^Ga-DOTATATE PET-CT scans were carried out on a Discovery STE dual PET-CT machine (GE Healthcare). The activity of ^68^Ga-DOTATATE injected intravenously was dependent on the yield from the ^68^Ga/Ge generator, but it was intended that 250 MBq be used. PET-CT scans were acquired after 45 min. No intravenous or oral contrast was used. A low-dose scout projection was used to localize the region of head to thigh for transmission and emission imaging*.* The low-dose CT component of the PET-CT scan was obtained at 120 kVp with modulated tube current (30–300 mA) and 1.75 pitch. CT slice thickness was 5 mm. In plane pixel resolution was 0.98 mm × 0.98 mm. A three-dimensional mode was used for PET acquisition, spending 4 min in each bed position. Images were iteratively reconstructed with 21 subsets with attenuation correction. PET-CT images were fused but were also available separately for analysis.

### PET-CT Image Analysis

#### Conventional PET-CT Analysis

Regions of interest (ROI) were drawn around the most avid lesions/metastases (up to five tumor foci) for each patient as seen on the ^68^Ga-DOTATATE PET-CT scan. For each patient within each lesion, standardized Gallium uptake value (SUV) on PET was quantified as the maximum (SUV_max_) and average (SUV_mean_) uptake values. Additionally, for each patient, the ^68^Ga-DOTATATE uptake tumor area on PET for each lesion was quantified as total number of pixels. This measurement is similar to the metabolic tumor area (which has increasingly been shown to be a useful prognostic biomarker ^18^F-FDG PET imaging), but the term “metabolic” in the setting of ^68^Ga-DOTATATE PET is a misnomer, hence using the descriptive term ^68^Ga-DOTATATE uptake tumor area.

#### CT and PET Texture Analysis (CT-TA and PET-TA)

Tumor heterogeneity was further assessed using texture analysis performed on the non-contrast, low-dose CT, as well as the attenuation-corrected PET components of the pretreatment ^68^Ga-DOTATATE PET scans, using a commercially available TexRAD research software (TexRAD, part of Feedback Medical Ltd, www.fbkmed.com, Cambridge, UK), which has been developed following a stringent regulatory and quality process based on ISO 13485 and 9001.

**Figure 1:**
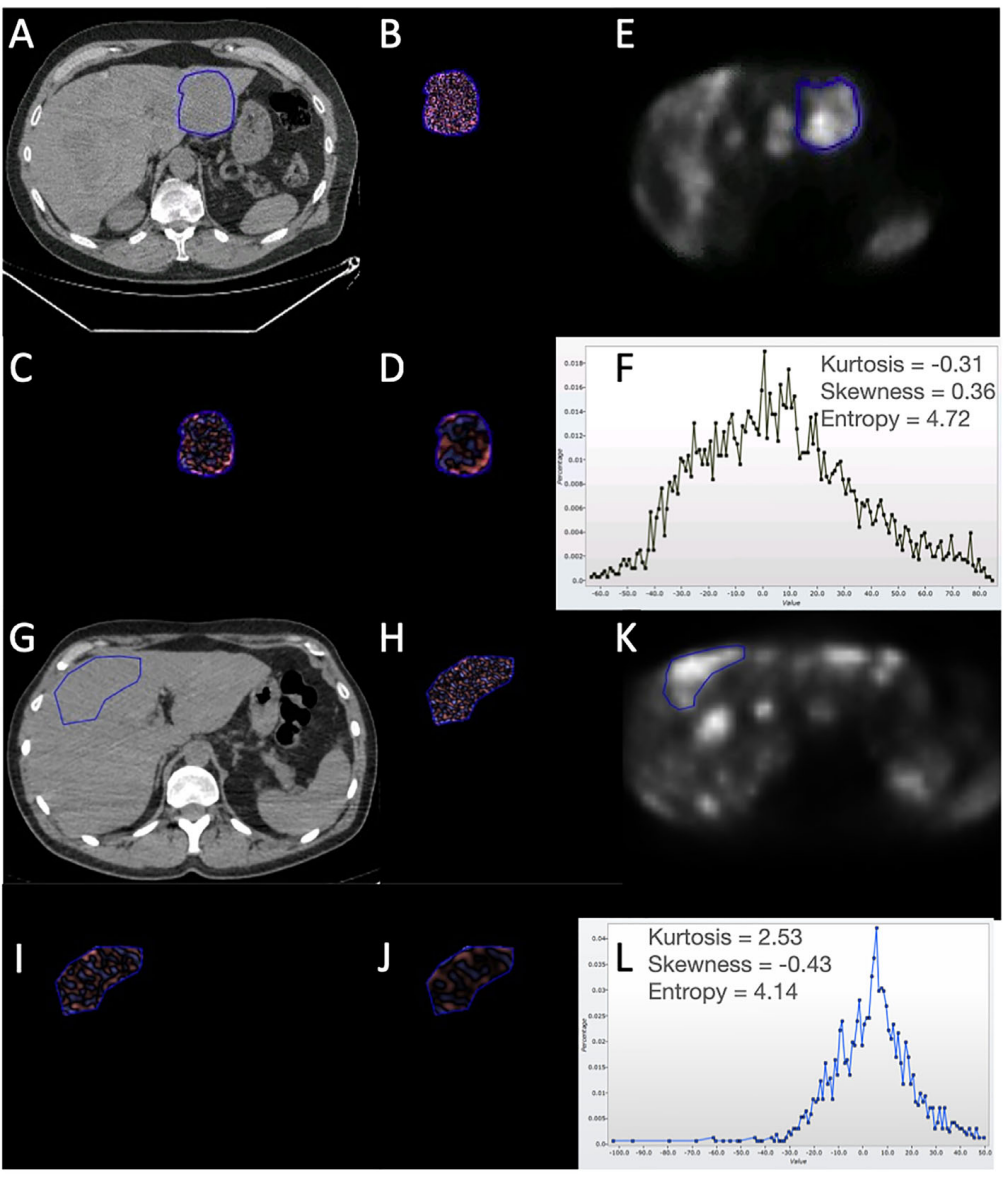
Illustration of the filtration-histogram-based texture analysis as applied on an individual liver lesion for two patients with NET on 68Ga-DOTATATE PET/CT. Top patient had a better response/outcome (PFS=30 months and OS=50 months) compared to the bottom patient (PFS=1 month and OS=6 months) to ^177^Lu-DOTATATE. **(A, G)** highlight the conventional CT image with the tumor encircled for the two patients, followed by corresponding **(B, H)**, which highlight the fine-texture map; **(C, I)** highlight the medium-texture map; **(D, J)** highlight the coarse-texture map; **(E, K)** highlight the PET image with the tumor encircled; and **(F, L)** highlight the histogram distribution along with the texture features (kurtosis, skewness, and entropy) for the coarse-texture map on CT (as an example). For the texture maps: Pink reflects positive filtered pixel intensity values, i.e., bright areas (the more intense the color, the brighter the area); blue reflects negative filtered pixel intensity values, i.e., dark areas (the more intense the color, the darker the area). For the PET-image: Brighter areas reflect increased avidity.

For each patient, two-dimensional ROIs were manually drawn around the individual lesions (identified on PET as described above) on the CT and PET slices corresponding to the most avid slice on PET. For CT-TA, each ROI underwent a filtration-histogram technique where the image filtration step (using a Laplacian of Gaussian band-pass filter, which is similar to a non-orthogonal Wavelet) extracted and enhanced objects/features of different sizes corresponding to different spatial scale filter (SSF), varying from 2 mm (fine), 3–5 mm (medium), and 6 mm (coarse) texture scale. This was followed by quantification using statistical and histogram-based analysis comprising mean gray-level intensity, standard deviation, entropy, mean of positive pixels, skewness, and kurtosis. Pixels within the ROI less than −50 HU (e.g., air, gases) were excluded from CT-TA. In addition, CT-TA also comprised of quantification without the use of image filtration as a control. In total, 36 texture features were quantified for CT-TA. Owing to the inherently low resolution associated with PET SUV images, PET-TA did not use image filtration which comprised six texture features. This filtration-histogram-based texture analysis methodology has undergone a qualification process demonstrating biological correlate, technical validation, clinical applications (prognosis, disease severity, treatment response/prediction), and cost-effective analysis evidenced through a number of publications (8–13). **Figure 1** provides an illustration of the filtration-histogram-based TA as applied on an individual lesion for a couple of patients with NET on ^68^Ga-DOTATATE PET/CT.

The texture analysis was conducted by a single radiographer (RE) with 20 years of experience in radiology and nuclear medicine under the supervision of a consultant radionuclide radiologist/nuclear medicine physician (SW) with 6 years of experience and senior imaging scientist (BG) with 12 years of experience in texture analysis. All of them were blinded to the clinical outcome.

### Clinical Parameters

Information about site of primary, location of metastases, and previous treatments was collected to assess the effect of clinical parameters on outcome measures.

### Statistical Analysis

For each patient the average value of the different image quantification from all available lesions was derived to provide patient level metrics. The relationships of tumor heterogeneity (quantified by CT-TA and PET-TA), Gallium DOTATATE uptake (SUV_max_, SUV_mean_, and ^68^Ga-DOTATATE uptake tumor area), and clinical markers with patient survival (PFS and OS) were assessed using univariate Kaplan-Meier (KM) survival analysis. Median values for these parameters were used as thresholds to separate the survival plots (poor and good prognostic groups). The significant differences in the survival plots were further evaluated using non-parametric log rank test. KM curves for patients above and below the median cut-off for the significant parameter were constructed.

A Benjamini-Hochberg correction using a false discovery rate of 30% was applied to the analysis in order to reduce the false discovery rate that can occur when making multiple comparisons (14).

Multivariate step-wise forward-Wald Cox regression analysis was used to determine which significant univariate markers (after Benjamini-Hochberg correction) were independent of each other in predicting patient survival along with the hazard ratio, 95% confidence interval. In all analyses, p values <0.05 were considered significant.

## Results

Forty-four patients were treated with ^177^Lu-DOTATATE between June 2007 and February 2016. Patient characteristics are listed in **Table 1**. Seventy-five percent of patients (33/44) received at least four treatments, and 18% (8/44) only had one dose of radionuclide.

**Table 1:** Patient characteristics.

	Number of patients (44)	%
**Gender**		
Female	15	34
Male	29	66
Median Age	56	
Age Range	28–87	
**Familial Syndrome**	**4**	**9**
SDHB mutation	3	7
MEN1	1	2
**Site of Primary Carcinoid**	**17**	**39**
- Lung	6	
- Small Bowel	6	
- Appendix	1	
- Large Bowel	3	
- Unknown Primary	1	
**Pancreatic NET**	**9**	**20**
**-** Gastrinoma	1	
- Glucagonoma	3	
- Insulinoma	1	
- Non-functioning	4	9
**Catecholamine secreting**	**7**	**16**
**-** Phaeochromocytoma	1	
- Paraganglioma	6	
**Thyroid**	**4**	**9**
**-** Hürthle Cell	1	
- Medullary Carcinoma	3	
**Head and Neck Adenocarcinoma**	**3**	**7**
**NET of Unknown Primary**	**4**	**9**
**Metastatic disease**	**44**	**100**
**-** Bone	23	52
- Brain	3	7
- Liver	29	65
- Lung	14	32
- Lymph Node	32	72
- Peritoneal	5	11
**Previous Treatments**	**44**	**100**
**-** Surgery	26	59
- PRRT	5	11
- RT	10	22
- Somatostatin Analogue	18	40
- Chemo	13	29
- Nil	4	9
**Treatments after Lu-DO**	**18**	**40**
**-** Surgery	2	5
- PRRT	3	7
- Somatostatin Analogue	2	5
- Chemo	7	16

The numbers in bold represent the number of patients for each main category.

The median follow-up time was 37 months (range 5–82 months), during which, 39 (80%) patients progressed and 28 (57%) patients died. The median PFS was 22 months (95% confidence interval: 10–34, IQR 8–40). The median OS was 37 months (95% confidence interval: 19–55, IQR 9–74).

Thirty-two patients had a ^68^Ga-DOTATATE PET-CT at 6 months after treatment to assess disease response. No patients had a complete response to ^177^Lu-DOTATATE; however, 10 patients (23%) had a partial response (PR), 20 had stable disease (SD) (63%), and 2 had disease progression (PD) (6%). Twelve (27%) patients had PD before 6 months and so did not have a response assessment scan.

### Univariate Kaplan-Meier Analysis

The PET-CT, texture, and clinical parameters that influenced PFS and OS at the median threshold values after Benjamini-Hochberg correction are detailed in **Tables 2** and **3**, respectively.

**Table 2:** Summary of univariate Kaplan-Meier analysis for PFS for the most significant CT and PET imaging parameters using the median value as the cut-off.

Imaging	Median cut-off (direction indicates poor prognosis)	Median survival in months (number of patients)		Kaplan-Meier p-value	Benjamini-Hochberg corrected p-value
		Below threshold	Above threshold		
**CT-TA**					
Kurtosis SSF 6	>0.29	35 (29)	14 (15)	0.049	0.05
**PET-TA (no filter)**					
Entropy	>3.79	39 (22)	12 (22)	0.0088	0.01
Skewness	<0.95	16 (37)	58 (7)	0.008	0.03
**PET conventional analysis**					
PET ^68^Ga-DOTATATE-uptake tumor area	>52.5 pixels	39 (22)	14 (22)	0.016	0.02
**Clinical parameters**					
Lung metastases	>1	35 (29)	14 (15)	0.026	0.04

**Table 3:** Summary of univariate Kaplan-Meier analysis for OS for the most significant CT and PET imaging parameters using the median value as the cut-off.

Imaging	Median cut-off (direction indicates poor prognosis)	Median survival in months (number of patients)		Kaplan-Meier p-value	Benjamini-Hochberg corrected p-value
		Below threshold	Above threshold		
**CT-TA**					
Kurtosis SSF 6	>0.29	50 (29)	16 (15)	0.022	0.05
**PET-TA (no filter)**					
Entropy	>3.79	53 (22)	22 (22)	0.0058	0.01
Skewness	<0.95	28 (37)	Not reached (7)	0.034	0.02
**PET conventional analysis**					
PET ^68^Ga-DOTATATE-uptake tumor area	>52.5 pixels	Not reached (22)	22 (22)	0.011	0.03
**Clinical parameters**					
Thyroid primary	>1	50 (44)	2(5)	0.013	0.04

#### Conventional PET-CT Analysis

**Figure 2:**
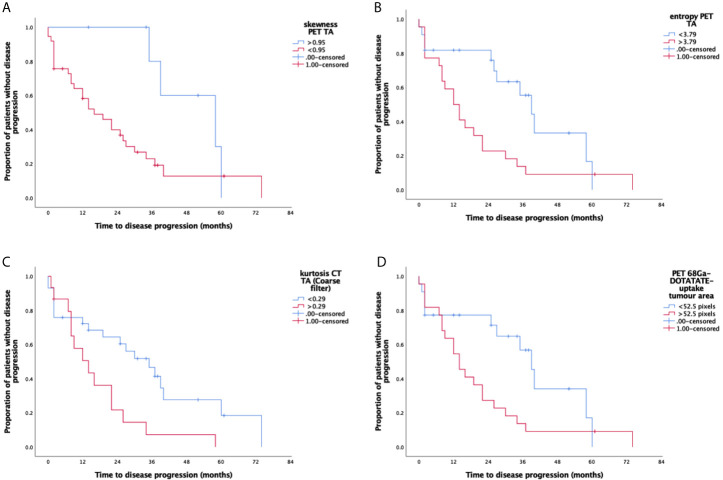
Kaplan-Meier survival curves for significant CT texture analysis (CT-TA), PET texture analysis (PET-TA), and PET uptake parameters against progression-free survival. Panel **(A)** PET-TA Skewness (without filtration), corrected p= 0.03, Panel **(B)** PET-TA Entropy (without filtration), corrected p=0.01, Panel **(C)** CT-TA Kurtosis (coarse), corrected p= 0.05, Panel **(D)** ^68^Ga-DOTATATE tumor uptake area, corrected p= 0.02. Median threshold values for each parameter are shown on the individual graphs.

**Figure 3:**
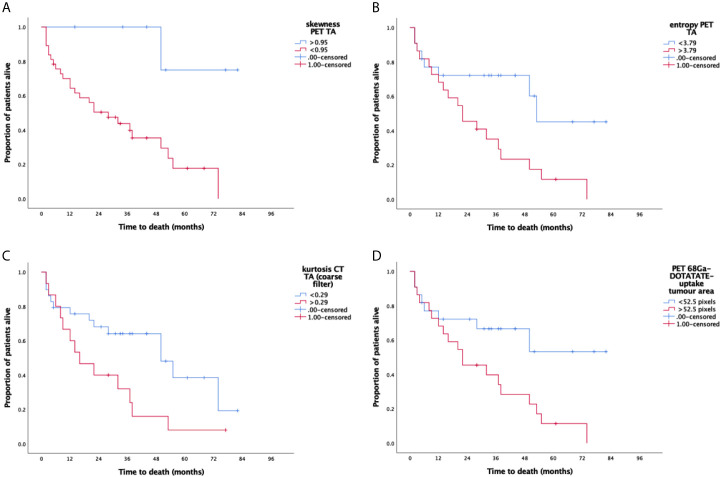
Kaplan-Meier survival curves for significant CT and PET texture parameters (CT-TA and PET-TA) against overall survival. Panel **(A)** PET-TA Skewness (without filtration), corrected p=0.02, Panel **(B)** PET-TA Entropy (without filtration), corrected p=0.01, Panel **(C)** CT-TA Kurtosis (coarse), corrected p=0.05, Panel **(D)** ^68^Ga-DOTATATE tumor uptake area, corrected p= 0.03. Median threshold values for each parameter are shown on the individual graphs.

Larger ^68^Ga-DOTATATE tumor uptake area was a significant marker of shorter PFS (p=0.016, **Table 2** and **Figure 2**) and OS (p=0.012, **Table 3** and **Figure 3**). Lower PET SUV_max_ and SUV_mean_ values demonstrated a trend towards shorter PFS and OS but did not reach statistical significance (PFS p=0.15 and p=0.17, respectively; and OS p=0.42 and p=0.33, respectively).

#### CT and PET Texture Analysis (CT-TA and PET-TA)

Among the different CT-TA parameters, higher kurtosis at coarse-texture scale (SSF=6 mm) was a significant marker of shorter PFS and OS [PFS p=0.049 (**Table 2**) and OS p=0.022 (**Table 3**)]. From among the different PET-TA parameters, higher entropy without filtration and lower skewness without filtration were significant markers of shorter PFS [entropy p=0.0088, skewness p=0.026 (**Table 2** and **Figure 2**)] and OS [entropy p=0.0058, skewness p=0.0073 (**Table 3** and **Figure 3**)].

#### Clinical Parameters

Of the different NET subtypes, patients with thyroid primaries had the worst survival with a mean OS of 6 months [p=0.013 (**Table 3**)]. Presence of lung metastases also negatively affected PFS [p=0.026 (**Table 2**)]. Otherwise, distribution of metastatic disease and previous treatments did not appear to have a significant effect on outcome in our cohort.

### Multivariate Cox Regression Analysis

When all of the significant predictors of PFS from the above univariate analysis (with Benjamini-Hochberg correction) were included in a multivariate Cox regression model, CT-TA kurtosis on coarse-texture scale (SSF 6) (HR=2.57, 95% CI=1.22–5.38, p=0.013) and presence of lung metastases (HR=2.97, 95% CI=1.37–6.46, p=0.006) independently predicted PFS. For OS, skewness on unfiltered PET-TA (HR=9.05, 95% CI=1.19–68.91, p=0.033) and having a thyroid primary (HR=6.21, 95% CI=1.92–20.12, p=0.002) were independent predictors on multivariate analysis.

## Discussion

The behavior of metastatic NET is varied as shown here where OS ranged between 0 and 82 months. TNM staging doesn’t distinguish between patients with one or many metastases, requiring other risk stratification techniques. Our study did not demonstrate that patients with bone metastases, functioning pancreatic NETs, or chemotherapy pretreatment fare worse, as have other series (15, 16). In this study, only a thyroid primary or lung metastases predicted poor survival. These inconsistencies highlight the need for other prognostic biomarkers.

Histological evaluation of the mitotic count and the Ki67 proliferation index are used to estimate aggressiveness, and these factors are crucial to WHO NET grading (17). However, histologically and phenotypically, there can be considerable heterogeneity between different metastatic deposits in one patient and within each focus of tumor (18, 19). Tumor de-differentiation may occur between presentation and recurrence, resulting in a discrepancy between disease behavior and the original histological appearances. Imaging assessments are time-specific and can assess all visible metastases.

We demonstrate how radiomics-based CT texture analysis could be used to assess the heterogeneity of metastatic NET to assist prognostication. Kurtosis on coarse filtered images and skewness and entropy on PET-TA were significantly related to survival outcomes. There is increasing interest in imaging of heterogeneity and radiomics-based texture analysis (20–25). Most of these studies focus predominantly on the PET-TA in NET demonstrating prognostic potential or treatment-related changes, where in one study (20) entropy significantly predicted outcome. However, our analysis, including both CT-TA and PET-TA, could be considered more comprehensive. Among all texture features analyzed in this research, kurtosis and entropy best distinguished between good and poor prognostic groups. These appear to consistently predict outcome in other TA studies and have been described as the most global measures of heterogeneity (13).

^68^Ga-DOTATATE PET has superseded octreoscan in being able to predict prognosis in NET (26). In several studies, SUV_max_ and SUV_mean_ have shown prognostic potential where higher values are associated with longer survival (2, 3). In this study, SUV_max_ and SUV_mean_ showed only trends towards predicting survival outcomes. The reliability of these parameters is questionable. For example, marked intra and interpatient variabilities in SUV values have been seen in studies reviewing quantitative SUV analysis in similar patient cohorts to ours (27). In the studies where higher ^68^Ga-DOTATATE avidity has predicted improved survival, there are large differences in the threshold values for SUV_max_ used in the literature, highlighting the potential for variability of this biomarker. SUV_max_ is a poor representation of the uptake in the tumor as a three-dimensional structure. We have described a new term, ^68^Ga-DOTATATE uptake tumor area (measured in pixels), because “metabolic” in this setting is not accurate. Larger ^68^Ga-DOTATATE uptake tumor area was significantly associated with both poor PFS and OS in the univariate analysis where SUV measures were not. MTV (metabolic tumor volume) has been explored in a number of cancers, with promising prognostic ability (28). Perhaps, with more validation, ^68^Ga-DOTATATE uptake tumor area could be used in a similar way.

CT and PET-TA is a developing technology. The filtration-histogram-based CT and PET-TA employed in this study has undergone rigorous qualification processes, essential for validating any biomarker. Miles et al. have highlighted how texture features extracted from the filtration-histogram technique reflect different components of heterogeneity (object/feature size, number, density in relation to the background tissue) (29). Furthermore, the texture features extracted from this filtration-histogram technique have been investigated in several tumor types where increased heterogeneity has been associated with inferior prognosis (8, 10–13, 30). To better understand NET, we need to correlate the imaging findings with histological parameters. In lung cancer, a number of texture parameters have been found to be associated with histological markers of angiogenesis and hypoxia (31). In a tumor where high Ki67 indicates de-differentiation and worse outcomes, it is plausible that the proliferation index would correlate well with heterogeneity on imaging. Conversely, SSTR2 expression, which is generally increased in well-differentiated tumors (32), would be expected to be inversely associated with tumor heterogeneity. We will investigate these associations in a future analysis, as well as the relationship between the noradrenaline transporter (NAT), which is overexpressed in catecholamine-secreting NETs, and TA.

### Limitations

It is important to acknowledge the significant drawbacks to retrospective research. There are several potential biases, including referral bias, selection bias, and information bias. We also acknowledge that the sample of patients is small, from a single center, and heterogenous including NETs with different biology and included patients who had been heavily pretreated. While this could be perceived to limit the interpretation of this study, in this preliminary, hypothesis-generating study, we chose to include all of these patients to reflect the variable NET population, allowing a practical review of TA. Moreover, due to the referral pathway of the majority of patients, we were unable to centrally review the histopathology and comment on Ki67 or grade of tumor in this cohort. We recognize that these parameters are prognostically important and plan to collect and analyze these data against TA in future prospective studies. Also, as the focus of this “theranostic” research study was to employ the radiomics analysis on the ^68^Ga-DOTATATE PET-CT scans as a prognostic marker in NET patients treated with ^177^Lu-DOTATATE, therefore only lesions that were avid on ^68^Ga-DOTATATE PET-CT were analyzed. In future studies we need to look at a range of lesions. Another limitation of the exploratory pilot study was to analyze only a single slice corresponding to the most avid part of the lesion. Single-slice analysis will potentially be time-efficient (within routine clinical practice) and potentially reduce operator variability (unless the segmentation is semi/fully automated) but may not entirely capture tumor heterogeneity information across the whole tumor volume, which will provide additional biological information potentially important in the clinical decision-making. Although previous studies, e.g., in PET/CT in lymphoma (33), have demonstrated the ability of single-slice texture analysis to provide prognostic information, we nevertheless believe that future studies should employ single-slice and volumetric analysis and compare both the approaches. In addition to the filtration-histogram-based texture analysis employed in this study, future work should evaluate a comprehensive radiomics analysis comprising of filtration-histogram, high-order statistics, and shape, along with their biological intuitiveness, inter- and intra-operator variability, and reproducibility assessment. Also, the use of a more sophisticated approach (e.g., cumulative assessment) for aggregating the individual lesion quantification rather than a simplistic approach (e.g., averaging) to get a patient-specific score needs to be evaluated in a future study. These are all essential for it to be used in clinical practice, so further prospective work is necessary in texture analysis on ^68^Ga-DOTATATE PET-CT in an independent, larger cohort including more clearly defined subclassifications of NETs and homogeneous treatment regime across several centers to validate our findings. If validated, radiomics-based TA in NET may have clinical value.

## Conclusion

We have demonstrated in this preliminary study that PET-CT-TA shows promise in the stratification of patients, and important prognostic information can potentially be obtained from one baseline scan. As patient outcomes are very variable, identification of those with the worst prognosis is important; they could be spared futile treatment and resources saved. Patients with more heterogeneous tumors, suggesting a more aggressive phenotype, might be offered chemotherapy earlier or could be considered for dual modality treatment with PRRT and chemotherapy as in the Australian Gastro-intestinal Trials Group study, CONTROL NETS.

## Data Availability Statement

Data not readily available due to institutional permissions, request to access dataset should be directed to the corresponding author, BG at b.ganeshan@ucl.ac.uk.

## Ethics Statement

Ethical approval for data collection and analysis was granted by HRA and Health and Care Research Wales (IRAS project number 204124). The patients/participants provided their written informed consent for treatment.

## Author Contributions

Guarantor of integrity of the entire study: MG and JB. Study concepts and design: CA, BG, MG, and JB. Literature research: CA and BG. Clinical Studies: CA, BG, RE, MA, and SW. Experimental Studies/Data analysis: CA, BG, RE, MA, SW, AG, and MG. Statistical Analysis: BG. Manuscript Preparation: CA, BG, and MG. Manuscript Editing: CA, BG, MG, and JB. All authors contributed to the article and approved the submitted version.

## Funding

This study was funded by the University College London Hospitals Charity, supported by the National Institute for Health Research University College London Hospitals Biomedical Research Centre. Dr M. N Gaze is supported by the Radiation Research Unit at the Cancer Research UK City of London Centre Award [C7893/A28990].

## Conflict of Interest

One author, BG, is a Co-Founder/Co-Inventor of TexRAD texture analysis software used in this study and a shareholder (not an employee) of Feedback Plc., a UK based company which owns, develops and markets the TexRAD texture analysis software.

The remaining authors declare that the research was conducted in the absence of any commercial or financial relationships that could be construed as a potential conflict of interest.

## Publisher’s Note

All claims expressed in this article are solely those of the authors and do not necessarily represent those of their affiliated organizations, or those of the publisher, the editors and the reviewers. Any product that may be evaluated in this article, or claim that may be made by its manufacturer, is not guaranteed or endorsed by the publisher.

## Abbreviations

NET, neuroendocrine tumor; TA, texture analysis; PET, positron emission tomography; CT, computed tomography; PFS, progression-free survival; OS, overall survival; ^68^Ga, ^68^Gallium; ^177^Lu, ^177^Lutetium; SUV, standardized uptake value; STTR, somatostatin receptor; PRRT, peptide receptor radionuclide therapy; KM, Kaplan Meier; IQR, interquartile range; PR, partial response; SD, stable disease; PD, progressive disease; SSF, spatial scale filter; MTV, metabolic tumor volume; NAT, nor-adrenaline transporter.
